# Effectiveness and Safety of Acupuncture and Moxibustion in Pregnant Women with Noncephalic Presentation: An Overview of Systematic Reviews

**DOI:** 10.1155/2019/7036914

**Published:** 2019-12-03

**Authors:** Maite Miranda-Garcia, Cristina Domingo Gómez, Cristina Molinet-Coll, Betina Nishishinya, Ikram Allaoui, M. Dolores Gómez Roig, Josefina Goberna-Tricas

**Affiliations:** ^1^Nursing and Health Ph.D Program, University of Barcelona, Barcelona, Spain; ^2^BCNatal. Barcelona Center for Maternal, Fetal and Neonatal Medicine, Sant Joan de Déu Hospital and Hospital Clínic, Barcelona, Spain; ^3^Institut de Recerca Sant Joan de Déu, Barcelona, Spain; ^4^Campus Docent Sant Joan de Déu, Barcelona, Spain; ^5^Quiron Traumatology Institute, Barcelona, Spain; ^6^Maternal and Child Health Development Network, RETICS. Research Institute Carlos III, Spanish Ministry of Economy and Competitiveness, Madrid, Spain; ^7^Department of Public Health, Mental Health and Perinatal Nursing, Faculty of Medicine and Health Sciences, Universitat de Barcelona, Barcelona, Spain; ^8^ADHUC Research Centre: Theory, Gender and Sexuality, Universitat de Barcelona, Barcelona, Spain

## Abstract

**Background:**

Breech presentation at the time of delivery is 3.8–4%. Fetuses that maintain a noncephalic presentation beyond 32 weeks will have a lower probability of spontaneous version before labor. Given the increasing interest in exploring the use of complementary medicine during pregnancy and childbirth, the moxibustion technique, a type of traditional Chinese medicine, could be another option to try turning a breech baby into a cephalic presentation.

**Objectives:**

To review the evidence from systematic reviews (SR) on the efficacy and safety of acupuncture and moxibustion in pregnant women with noncephalic presentation.

**Main Results:**

Our SR synthesizes the results from five clinical trials on pregnant women with a singleton noncephalic presentation. There is evidence that moxibustion reduces the number of noncephalic presentations at the time of birth compared with no treatment. The adverse effects that acupuncture and moxibustion can cause seem to be irrelevant. Most SRs agree that there are no adverse effects directly related to acupuncture and moxibustion.

**Conclusions:**

Even though the results obtained are positive and the five reviews conclude that moxibustion reduces the number of noncephalic presentations at birth (alone or combined with postural techniques or acupuncture), there is considerable heterogeneity between them. Better methodologically designed studies are required in the future to reaffirm this conclusion.

## 1. Introduction

The incidence of breech presentation at the time of delivery is 3.8–4% [1]. If this situation is not solved, the incidence of elective cesarean section increases. Hannah et al. demonstrated a reduction of infant morbidity and mortality with elective caesarean birth compared with vaginal birth in fetuses with breech presentation [2].

The causes of breech presentation had been extensively studied, and it is associated with maternal age, female fetuses, prematurity, small-for-gestational-age fetuses, congenital defects, multiple gestations, primiparity and multiparity, uterine malformations, placenta previa, and oligohydramnios [3]. Fetuses with a breech presentation after 32 weeks will have a lower probability of spontaneous cephalic version before birth.

It is a known fact shared among health professionals that birth by cesarean section increases maternal and perinatal morbidity and mortality compared with vaginal birth. The incidence of severe obstetric morbidity is between 0.05% and 1.09% [4, 5], and maternal mortality after a caesarean section is six times higher than that after a vaginal delivery. Therefore, complications during and after caesarean section are potentially serious and increase the risk of maternal, fetal, and neonatal mortality [6].

Furthermore, a cesarean section represents significant financial expenditure for health services [7]. In recent years, governments, public authorities, and health professionals had shown their concern regarding the increase of caesarean births, with their reduction becoming a priority objective [8]. This situation is accentuated by the rise in days of hospitalization in the case of caesarean delivery, compared with vaginal birth without complications, the former being double even with a normal course. However, the consequences of cesarean section are not only financial but also psychological because many patients experience it as a forced and traumatic intervention [9, 10].

One solution, before delivery, is external cephalic version (ECV), by which the fetus is turned to cephalic presentation. This noninvasive maneuver consists of manipulating the fetus through the maternal abdomen, requiring specific conditions to promote the change in fetal presentation: hospital environment, trained personnel, and simultaneous ultrasound. There are also anatomical factors that simplify the ECV, such as posterior placenta and a normal volume of amniotic fluid. The effectiveness of this maneuver is about 50–80% and severe complications are very low [10].

Given the growing interest in complementary medicine during pregnancy and delivery, the moxibustion technique, originating in traditional Chinese medicine, could be another option for trying to switch breech to cephalic presentation.

Moxibustion consists of burning of a herb (*Artemisa vulgaris*) close to the skin to induce heat at acupuncture point bladder 67 (BL67) [11]. The acupuncture point BL67 (Chinese name *Zhiyin*), located on the tip of the fifth toe, could fix breech presentation [12]. It is thought that moxibustion stimulates estrogen and prostaglandin production, increasing fetal activity and uterus contraction [13]. In addition to moxibustion, acupuncture could be used for this or other points with similar results [14].

The objective of this review is to explore the evidence from systematic reviews (SRs) on the efficacy and safety of acupuncture and moxibustion in pregnant women with noncephalic presentation.

## 2. Methods

### 2.1. Search Strategy and Selection Criteria

Systematic reviews were identified and included through a sequential search procedure of the following databases: Medline (2014 to June 2018), the Cochrane Central Register of Controlled Trials (CENTRAL, 2012 to June 2018), Work Science (2016 to June 2018), and CINHAL (2015 to June 2018).

The search terms used were “breech presentation,” “moxibustion,” “acupuncture,” “moxa,” “artemisia,” “obstetrics,” “noncephalic presentation,” “presentación de nalgas,” “presentación podálica,” “moxibustión,” “acupuntura,” “moxa,” “artemisa,” “obstetricia,” and “presentación no cefálica.”

The Boolean combinations were the following: “moxibustion AND breech presentation,” “artemisa AND breech presentation,” “acupuncture OR moxibustion AND breech presentation,” and “artemisa OR moxibustion AND presentation noncephalic.”

Only articles in English and Spanish were included.

## 3. Inclusion Criteria

### 3.1. Types of Studies

Only SRs of randomized and cohort studies related to pregnant women with a single fetus and noncephalic presentation were included.

### 3.2. Types of Intervention

We included reviews on the effectiveness of treatments for the version of the fetus in noncephalic presentation. The intervention methods included moxibustion, moxibustion plus acupuncture, moxibustion plus postural techniques, acupuncture, electroacupuncture, laser stimulation, and ear acupuncture.

## 4. Exclusion Criteria

Other types of study designs.

### 4.1. Quality of Studies

**Figure 1:**
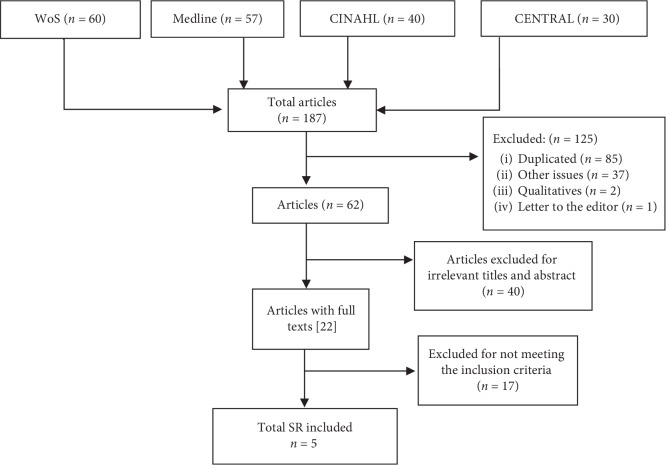
Flow diagram for selecting systematic review literature on the effectiveness and safety of acupuncture and moxibustion in pregnant women with noncephalic presentation.

All the potential studies identified were independently assessed by two review authors independently (MM, CD) following the AMSTAR (A Measurement Tool to Assess Systematic Reviews) quality assessment instrument. AMSTAR is an 11-item instrument that assesses the quality of systematic reviews and internal and external validity [15, 16]. Discrepancies were resolved through discussion (see Figure 1).

## 5. Search Strategy for the Identification of Studies and Selection of Systematic Reviews

### 5.1. Characteristics of the SRs

Five SRs included Coyle et al. [17], Van den Berg et al. [18], Vas et al. [19], Li et al. [20], and Zhang et al. [21]. The research identified six reviews; however, the Coyle et al. review [22] was excluded as it was updated in the 2012 version [17]. In general, these SRs reported 24 primary studies, 17 of which were randomized clinical trials (RCTs) and 7 cohort studies (CCT). It should be noted that the quality of the reviews was high (AMSTAR 7–11) (Table 1). There were a total number of 5,339 pregnant women with a noncephalic presentation. The five systematic reviews were published in English (see characteristics of the studies in Tables 2 and 3).

## 6. Results

### 6.1. Type of Delivery

Two of the SRs [17, 18] report the implications for future research, taking measures of clinically relevant outcomes such as mode of birth and neonatal outcome, which includes safety, morbidity, and mortality.

### 6.2. Cesarean Rate

In the Coyle et al. SR [17], the result obtained was that with the use of moxibustion compared with no treatment there are fewer cesarean births (RR 0.79, 95% CI 0.64 to 0.98). Two studies reported a favorable number of cesarean births [23, 24]. A meta-analysis showed no difference in the rate of cesareans between the treatment and control groups (RR 1.05, 95% CI 0.87 to 1.26), but the results of this meta-analysis should be considered with caution, due to clinical heterogeneity.

The SRs highlight that in the Neri et al. [25] trial, which performed moxibustion plus acupuncture versus no treatment, there was a lower rate of cesarean sections in women (RR 0.79, 95% CI 0.64 to 0.98). In the Van den Berg et al. SR [18], the cesarean rate is described in the three primary studies included [18, 26, 27].

Some of the studies selected by Van der Berg et al. [18] consider cesarean section an important result to assess the effect of treatment. However, she points out that the number of cesarean sections performed is determined by the effect of the treatment and by other factors, such as other medical indications and the women's own preferences. In other studies included in this review, cesareans were not studied as a result. In two SRs [19, 20], the number of cesarean sections is not mentioned as a noteworthy fact.

In the Zhang et al. SR [21], there are three studies [23, 24, 28] that consider the number of cesarean sections performed as a result.

### 6.3. Measurement of Oxytocin Levels

There are two SRs that refer to the measurement of oxytocin as a parameter to assess the effectiveness of the moxibustion intervention.

Coyle et al. [17] shows that moxibustion compared to no treatment allows a decrease in the use of oxytocin before or during labor in women with vaginal deliveries (RR 0.28, 95% CI 0.13 to 0.60), in Cardini and Weixin [23]. Also, in the Zhang et al. SR [21] significant differences were found in favor of a reduced use of oxytocin in the treatment group (RR 0.28, 95% CI 0.13 to 0.60).

### 6.4. Adverse Events

In the five SRs [17–21], the study by Cardini and Weixin [23] stands out as it reports intrauterine fetal death in the control group (RR 0.33, 95% CI 0.01 to 8.11), but indicates that no adverse events occurred during the treatment. However, it refers to three cases of premature membrane rupture at 37 weeks, two weeks after the end of treatment.

Coyle [17] also reports that women in the treatment group reported an unpleasant odor, with or without throat problems, due to the discomfort of moxa smoke and comments on the possibility of nausea and abdominal pain from contractions. This review mentions the study by Neri et al. [25] that measures the heart rate and blood pressure of the pregnant woman and the fetal heart rate immediately after the intervention, with no changes detected. It also noted that preterm uterine contractions were not detected.

Van der Berg et al. [18] also reports respiratory problems due to moxa smoke and comments that uterine contractions and hypertension may appear.

Vas et al. [19] reports that there is a trend toward fewer complications in the treatment group.

The Zhang SR [21] referring to the Do et al. study [28] reports two cases of premature labor and three cases of premature membrane rupture. He also comments that no statistically significant differences were found between the moxibustion group and the nonmoxibustion group in the values for Apgar at 7 to 5 minutes after cesarean section, premature delivery, membrane rupture, intrauterine fetal death, detachment of placenta, and umbilical cord blood pH under 7.1.

## 7. Discussion

The systematic reviews included for analysis were carried out between 2008 and 2013; this indicates a growing interest in this topic.

The sample sizes vary considerably, making it difficult to reach definitive conclusions on the results. It should be noted that the number of pregnant women in the study groups varied greatly, from 20 pregnant women [28] in the study conducted in Australia to a sample of 820 pregnant women [29] in the study conducted in China. This larger sample number may be due to the fact that moxibustion is a well known form of treatment with greater acceptance in Asian countries compared with Western countries.

The objectives of the five SRs are not homogeneous, but coincide in pointing out the effectiveness and safety of moxibustion in all of them, except for one [18] that only assessed effectiveness.

We found seven different treatment techniques for noncephalic presentation, the most used and effective being moxibustion in BL67 in the intervention group.

It is also important to assess the frequency of treatment. Four systematic reviews [17–19, 21] report the existence of great variability among those who perform the treatment once or twice a day or once or twice a week. Only Li et al. [20] did not mention this result. The period is also variable in all studies, ranging from 3, 7, or 10 consecutive days of treatment to 1, 2, or 3 weeks and up to 40 days.

Such variability in frequency of application of treatment and in the treatment period makes it difficult to obtain reliable and extrapolatable results. According to the SR [18], the most common frequency is two sessions per week for two weeks of treatment.

Another parameter cited in two SRs [18, 21] is oxytocin measurement. They observed that oxytocin use at delivery decreased in the treatment group.

One SR [17] shows that cesarean deliveries were lower in the treatment group while a further SR [18] considers cesarean section an important result in assessing the effectiveness of treatment. However, it is worth noting that the number of cesarean sections was determined by the effect of treatment and also by other factors, such as other medical indications and women's preferences.

The adverse events identified in the different reviews seem not to be relevant. All the systematic reviews agreed that there are no adverse effects directly related to moxibustion or acupuncture technique, so it seems to be safe. Some of the described effects were of little importance and not all the studies commented on the adverse effects.

### 7.1. Implications for Practice

Coyle [17] concludes that there is evidence to suggest moxibustion can reduce the number of noncephalic presentations at birth, either alone or in combination with acupuncture or postural techniques. The other reviews do not include a section on implications for practice, although they refer to them in other terms.

It would be interesting to include measurement of oxytocin as a parameter in the studies, because its use seems to decrease in pregnant women with treatment; however, more evidence is needed to confirm or reject the benefits.

As secondary outcomes, it would be interesting to have data regarding the type of delivery and neonatal outcomes.

In addition to performing the moxibustion treatment, if the fetus has not turned to a cephalic presentation, the external cephalic version or postural techniques could be performed, thereby contributing to increasing cephalic presentations.

According to the recommendations in Coyle [14], for future research studies should report the safety and also pregnant women's opinions on the intervention. He also highlights the importance of exploring the number of sessions, the frequency, and duration of treatment.

Although the results obtained in all reviews are positive, more studies are needed in the future to establish clearer protocols for the efficacy, effectiveness, and safety of moxibustion for pregnant women with noncephalic presentation.

### 7.2. Review Limitations

Van den Berg et al. [18] proposes the design of an ideal study, which would be a placebo-controlled RCT. However, a relative contraindication to the use of moxibustion during pregnancy at a location other than BL67 is mentioned, which would not allow the use of moxibustion treatment as placebo. Coyle et al. [17] also mentions that blinding the participants is not currently achievable, so comparison of moxibustion with ECV or postural techniques is suggested.

Not all SRs were able to perform a meta-analysis due to the considerable heterogeneity in the results; thus, it was not possible to obtain conclusive data.

### 7.3. Implications for Research

Coyle et al. [17] refers to the need for a robust methodology, with adequate statistical power to evaluate each intervention. Parity, gestational age at the time of intervention, and ethnicity should be taken into account as important variables.

According to the review by Li et al. [20], there is a lack of multicenter studies to be able to offer more evidence.

According to Zhang et al. [21], more rigorous high-quality RCTs are needed in the future to evaluate the safety and efficacy safety of moxibustion for correcting noncephalic presentation.

One of the SRs [20] reports that qualitative research can help interpret the research findings. Therefore, more trials are needed to address other aspects such as the study environment and preferences and expectations among pregnant women.

## 8. Conclusions

The five SRs concluded that moxibustion alone or in combination with acupuncture or postural techniques can reduce the number of noncephalic presentations at birth.

The results should be interpreted with caution. More studies are needed with a better designed methodology to assess this intervention. There are numerous differences in the trial designs, resulting in considerable heterogeneity.

It also seems to be the case that the moxibustion intervention for noncephalic presentation decreases the oxytocin use compared with no treatment.

It should be noted that blinding participants to assign them moxibustion is not feasible, so it would be useful to design clinical trials in the future that compare the moxibustion group to an external cephalic version to provide better results.

## Figures and Tables

**Table 1:** Methodological quality of systematic reviews included in moxibustion for pregnant women with a noncephalic presentation.

Author (year)	1	2	3	4	5	6	7	8	9	10	11
Coyle 2012 [17]	Y	Y	Y	Y	Y	Y	Y	Y	Y	Y	Y
Li 2009 [20]	Y	Y	Y	NA	Y	Y	Y	NA	N	N	Y
Van den Berg 2008 [18]	Y	Y	Y	Y	Y	Y	N	Y	Y	N	Y
Vas 2009 [19]	Y	Y	Y	N	Y	Y	Y	Y	Y	Y	N
Zang 2013 [21]	Y	Y	Y	Y	Y	Y	Y	Y	Y	Y	Y
# Yes (%)	5 (100)	5 (100)	5 (100)	3 (60)	5 (100)	5 (100)	4 (80)	4 (80)	4 (80)	3 (60)	4 (80)

N no; NA, not applicable; NR, not reported; Y yes (systematic review fulfilling the criteria); # of Yes, number of yes; AMSTAR item: 1. Was an “a priori” design provided? 2. Was there duplicate study selection and data extraction? 3. Was a comprehensive literature search performed? 4. Was the status of publication (i.e. grey literature) used as an inclusion criterion? 5. Was a list of studies (included and excluded) provided? 6. Were the characteristics of the included studies provided? 7. Was the scientific quality of the included studies assessed and documented? 8. Was the scientific quality of the included studies used appropriately in formulating conclusions? 9. Were the methods used to combine the findings of studies appropriate? 10. Was the likelihood of publication bias assessed? 11. Was the conflict of interest included? [15].

**Table 2:** Characteristics of the studies.

Study (year)	Included studies	Meta-analysis	Objectives	Sample size
Coyle et al. [17] (2012)	8 RCTs	Yes	To examine the safety and efficacy of moxibustion in breech presentation, the need for external cephalic version, mode of birth, and perinatal morbidity and mortality	1346

Li et al. [20] (2009)	10 RCTs 7 CCTs	Yes	To assess the safety and efficacy of moxibustion and other methods of stimulating acupuncture points to treat breech presentation in pregnant women	2090

Van den Berg et al. [18] (2008)	6 RCTs 3 CCTs	No	To evaluate the efficacy of the interventions (moxibustion, acupuncture, or electroacupuncture) in acupuncture point V67 for the presentation of breech, in comparison with the expectant behavior	1624

Vas et al. [19] (2009)	7 RCTs	Yes	To demonstrate the safety and efficacy of moxibustion, compared with a control (expectant management, postural methods, or acupuncture) to correct the noncephalic presentation	1067

Zhang et al. [21] (2013)	7 RCTs	Yes	To evaluate the safety and efficacy of moxibustion for the correction of noncephalic presentation	1387

**Table 3:** Characteristics of studie II.

Study (year)	Intervention	Control	Results	AMSTAR Score
Coyle et al. [17] (2012)	Moxibustion Moxibustion in combination with postural techniques Moxibustion in combination with postural techniques	Moxibustion Acupuntura Buttocks Knee-chest position	Moxibustion produces fewer noncephalic presentations at birth compared with acupuncture (RR 0.25, 95% CI 0.09 to 0.72) The combination of acupuncture and moxibustion is effective for cephalic version at birth (RR 0.73, 95% CI 0.57 to 0.94) and has fewer cesarean births (RR 0.79, 95% CI: 0.64 to 0.98) Moxibustion combined with postural techniques resulted in fewer noncephalic presentations at birth (RR 0.26, 95% CI 0.12–0.56)	11/11

Li et al. [20] (2009)	Moxibustion Acupuncture Electroacupuncture Laser stimulation Ear acupuncture	No treatment No treatment or knee-chest position Knee-chest position Raising buttocks method Knee-chest position plus pelvis rotating	Meta-analysis showed significant differences between moxibustion and no treatment (RR 1.35, 95% CI 120–155, 3 RCT) Moxibustion compared to the knee-chest position did not show significant differences (RR 1.30, 95% CI 0.95 to 1.79, 3 RCT) Moxibustion plus other therapeutic methods showed significant beneficial effects (RR 1.36, 95% CI 1.21–1.54, 2 RCT) Laser stimulation was more effective than the thorax-knee position plus pelvis rotation Moxibustion was more effective than other treatments (RR 1.29, 95% CI 1.17–1.42, 2 CCT) with the exception of the knee-knee treatment (RR 1.22, 95% CI 1.11–1.34, 2 CCT) Laser stimulation in Zhiyin (BL 67) was more effective than treatment with the knee-chest position (RR 1.30, 95% CI 1.10–1.54, 2 CCT)	7/11

Van den Berg et al. [18] (2008)	Moxibustion Moxibustion in combination with acupuncture Electroacupuncture stimulation	Does not specify	The pooled RCTs demonstrated a significant effect from the intervention, the proportion of breech presentations in the intervention group was 34% versus 66% in the control group (OR 0.25, 95% CI 0.11–0.58) In the pooled controlled cohort series, the proportion of breech presentations in the intervention group was 15% (*n* = 347) versus 36% in the control group (*n* = 459) (OR 0.29, 95% CI 0.19–0.43) Combining all studies, the proportion of breech presentations in the intervention group was 28% (*n* = 768) versus 56% in the control group (*n* = 856) (OR 0.27, 95% CI 0.15–0.46)	9/11
Vaset al. [19] (2009)	Moxibustion Acupuncture Postural methods Version cephalic extern	Observation Knee-chest position Bilateral acupuncture at BL 67	Moxibustion (alone or in association with acupuncture or postural measures) with mere observation or postural measurements showed a high degree of heterogeneity and a cephalic version rate in the moxibustion group of 72.5% vs. 53.2% in the control group (RR, 1.36, 95% CI, 1.17–1.58) An analysis was carried out to compare moxibustion and acupuncture, with a single study available that reported a cephalic version rate of 80% in the moxibustion group vs. 28% in the acupuncture group (RR, 4.0; 95%, 1.13–14.17)	9/11

Zhang et al. [21] (2013)	Moxibustion Knee-chest therapy	Knee-chest therapy Observation Usual antenatal care for Expectant management	The meta-analysis of 4 studies showed encouraging effects in favor of moxibustion in cephalic presentation at delivery (excluding ECV) (RR 1.29, 95% CI, 1.22–1.49) The same findings were applied to cephalic presentation after cessation of treatment when moxibustion (alone or combined with postural techniques) was compared with observation or postural technique (RR 1.36, 95% CI, 1.42–1.86)	11/11
